# An Experimental Feasibility Study Evaluating the Adequacy of a Sportswear-Type Wearable for Recording Exercise Intensity

**DOI:** 10.3390/s22072577

**Published:** 2022-03-28

**Authors:** Yoshihiro Marutani, Shoji Konda, Issei Ogasawara, Keita Yamasaki, Teruki Yokoyama, Etsuko Maeshima, Ken Nakata

**Affiliations:** 1 Graduate School of Sport and Exercise Sciences, Osaka University of Health and Sport Sciences, Kumatori 590-0496, Osaka, Japan; marutani@mspa.med.osaka-u.ac.jp (Y.M.); etsukom@ouhs.ac.jp (E.M.); 2 Department of Health and Sport Sciences, Osaka University Graduate School of Medicine, Toyonaka 560-0043, Osaka, Japan; skonda@caos.med.osaka-u.ac.jp (S.K.); ogasawaraissei@hss.osaka-u.ac.jp (I.O.); yamasaki@gim.med.osaka-u.ac.jp (K.Y.); tyokoyam@cardiology.med.osaka-u.ac.jp (T.Y.); 3 Department of Sports Medical Biomechanics, Osaka University Graduate School of Medicine, Suita 565-0871, Osaka, Japan

**Keywords:** wearable sensor, acceleration, electrocardiogram, heart rate, exercise intensity

## Abstract

Sportswear-type wearables with integrated inertial sensors and electrocardiogram (ECG) electrodes have been commercially developed. We evaluated the feasibility of using a sportswear-type wearable with integrated inertial sensors and electrocardiogram (ECG) electrodes for evaluating exercise intensity within a controlled laboratory setting. Six male college athletes were asked to wear a sportswear-type wearable while performing a treadmill test that reached up to 20 km/h. The magnitude of the filtered tri-axial acceleration signal, recorded by the inertial sensor, was used to calculate the acceleration index. The R-R intervals of the ECG were used to determine heart rate; the external validity of the heart rate was then evaluated according to oxygen uptake, which is the gold standard for physiological exercise intensity. Single regression analysis between treadmill speed and the acceleration index in each participant showed that the slope of the regression line was significantly greater than zero with a high coefficient of determination (walking, 0.95; jogging, 0.96; running, 0.90). Another single regression analysis between heart rate and oxygen uptake showed that the slope of the regression line was significantly greater than zero, with a high coefficient of determination (0.96). Together, these results indicate that the sportswear-type wearable evaluated in this study is a feasible technology for evaluating physical and physiological exercise intensity across a wide range of physical activities and sport performances.

## 1. Introduction

The benefits of wearable sensors have attracted attention worldwide. With the ability to monitor the intensity of physical activity in real time, the field of sports medicine has recognized that wearables are technically superior to conventional monitoring techniques, such as subjective questionnaires [1,2,3,4]. Wearables thus allow exercise management and training programs to be quantitatively analyzed [5,6,7]. Hence, most wearables today use a combination of physical and physiological indices. The former evaluates physical intensity, determined using a global positioning system (GPS) and accelerometer to quantify displacement, speed, and acceleration [8,9,10]. The acceleration is recorded easily by small inertial sensors, and acceleration-derived indices (magnitude of tri-axial acceleration or its cumulative value) are used to determine physical intensity [11]. Conversely, the heart-rate-derived indices (percentage of maximum heart rate and heart rate reserve) represent physiological intensity [12,13]. Together, these indices allow exercise intensity to be measured. Further, the effects of exercise have traditionally been measured via oxygen uptake [14]; however, this monitoring method is not suitable for non-experimental settings. Alongside the rise in the use of wearables, concurrent evaluation of physical and physiological intensity, in which acceleration-derived indices and heart-rate-derived indices have become the preferred monitoring, enables non-invasive, day-to-day observations [15,16,17].

The most popular consumer wearable is the watch-type sensor worn on the wrist, which allows the physical and physiological indices of daily living, exercise, and sports activity to be recorded [18,19]. The watch-type wearable measures the heart rate, which is estimated by the change in blood flow recorded by the photoplethysmography [20]. The watch-type wearables have been widely used in track-and-field competitive sports and others, as well as daily health monitoring and recreational sports. However, the watch-type wearables cannot be used in exercise and sports that may involve contact with opponents. Another wearable is the chest-belt-type, which has been used in experimental environments and competition fields [21,22]. The heart rate is determined by the ECG recorded by the electrodes embedded in the chest belt, which allows the accurate determination of heart rate the same as clinical evaluations [23,24,25]. A possible disadvantage of the chest-belt-type wearable is that it has been known to be a discomfort due to the wrapping around the chest [26].

Recently, a sportswear-type wearable with a small inertial sensor attached to the chest and two ECG electrodes sewn on opposite sides has been developed [27] as a new type of wearable, and some devices have been commercially available. A major advantage of sportswear-type wearables is that they are as comfortable as sportswear, which enables athletes to wear them in daily training, practice, and even in competitive matches. In addition to the existing watch- and chest-belt-types, sportswear-type wearables will be expected to contribute as a new device to increase the variety and choice of wearable, enabling the monitoring of exercise intensity during competitive matches and daily practice for athletes. A previous study had only demonstrated the feasibility of recording ECG signals by the sportswear-type wearable while running [28,29]; however, the concurrent feasibility of evaluating physical and physiological intensities using a sportswear-type wearable is not yet known. With our study, we aimed to examine this across a wide range of physical activities, within a controlled laboratory setting.

## 2. Methods

### 2.1. Participants

Six male college athletes (mean age ± SD: 18.8 ± 0.4 years; mean height ± SD: 171.2 ± 7.1 cm; mean weight ± SD: 62.2 ± 5.5 kg; mean BMI ± SD: 19.1 ± 4.0 kg/m^2^) volunteered to participate in this study. There was only one type of the examined sportswear-type wearable, and it was fitted sufficiently for males but insufficient fit for females. This study was conducted for only male participants. Inclusion criteria for the study were the following: (1) collegiate male athletes who exercised regularly; (2) were disease-free and in good health; (3) had previous experience with a treadmill; (4) were able to complete the measurement protocol. Prior to participating in the study, each volunteer provided written informed consent, and the study was approved by the Institutional Review Board (observation research ethics review committee of the Osaka University Hospital: 19537-2).

### 2.2. Data Collection

**Figure 1:**
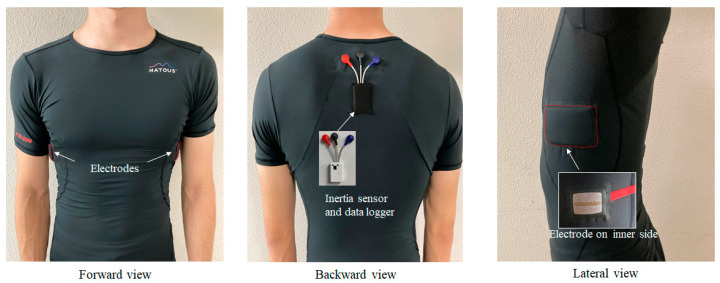
Sportswear-type wearable examined in this study. ECG electrodes are located under each armpit, and the inertial sensor embedded into the data logger is mounted on the upper back.

Each participant was asked to wear an elastic sportswear-type wearable (MATOUS, Teijin Frontier Ltd., Osaka, Japan) that is similar in comfort to exercise underwear. The material of sportswear-type wearable is polyester and nylon material, and the electrode part is constructed of polyester and conductive yarn. Prior to the experiment, the collegiate athletes confirmed that they felt no discomfort wearing it during sports activities. A data logger operating at 1000 Hz (SS-ECGHRAG, Sports Sensing Ltd., Fukuoka, Japan) was also attached to each participant’s upper back. An inertial sensor was built into the data logger (Figure 1), while two ECG electrodes were located on either side of the wearable. Both the ECG and acceleration signals were recorded by the data logger and transmitted to a laptop computer. Using a respiratory gas analyzer (AE-300S AEROMONITOR, Minato Medical Science Ltd., Tokyo, Japan), the breath-by-breath method was implemented to measure oxygen uptake (${\dot{\mathrm{Vo}}}_{2}$), which is the gold standard physiological index. This allowed us to examine the feasibility of using the heart rate obtained by the sportswear-type wearable as an indicator of exercise intensity. A linear relationship has been reported between heart rate and oxygen uptake [16]; therefore, the use of oxygen uptake, which is the gold standard for evaluating physiological intensity, is appropriate for a feasibility study.

A linear incremental loading test was performed using a treadmill (Elite T5000, Johnson Health Tech Japan Ltd., Osaka, Japan) set at a zero-degree incline. After recording the measurement at rest (standing), the participants were made to walk (1–6 km/h), jog (7–12 km/h), and run (13–20 km/h); speed was increased by 1 km/h every 30 s. All measurements were taken in an indoor laboratory. This allowed us to examine the mechanical feasibility of evaluating the acceleration index under an exercise, intensity-controlled, condition.

### 2.3. Data Analysis

The acceleration’s x-axis, y-axis, and z-axis were measured by the inertial sensor and then filtered by a Butterworth band-pass filter (0.5 Hz–20 Hz) to remove the high-frequency noise and the baseline shift, and then the norm of acceleration ($\left|a\right|=\sqrt{a_{x}^{2\;}+a_{y}^{2\;}+a_{z}^{2\;}}$) was calculated. Then, the norm of acceleration was filtered by a moving average filter with a 1 s window, defined as acceleration index. Following a Butterworth high-pass filtering process with a cutoff frequency of 5 Hz, the ECG signal was also filtered using a fourth order Savitzky–Golay filter [30,31]. The filtered ECG data were used to detect the R-R interval and calculate the heart rate per minute (bpm). Then, the calculated heart rate was filtered by a moving average filter with a 1 s window. Oxygen uptake was averaged every 30 s using the respiratory gas analyzer software on the output of each breath, from rest to exercise completion. Data analysis was then performed using a custom-made MATLAB R2020a program (MathWorks, Inc., Natick, MA, USA).

### 2.4. Statistical Analysis

Heart rate, acceleration index, and oxygen uptake during the last stable 15 s were averaged and used as representative values for each participant within each experimental condition. The mean values and standard deviations across all participants were determined using descriptive statistics. The relationship between heart rate and oxygen uptake was determined using a single regression analysis. To examine whether the acceleration index is equivalent to a change of 1 km/h on the treadmill or if the index adequately reflects the change in exercise intensity, single regression analysis between acceleration indices and treadmill speeds was performed for each participant, for the following exercise types: low-intensity (walking: 1–6 km/h); medium-intensity (jogging: 7–12 km/h); and high-intensity (running: 13–20 km/h). We assumed that the acceleration index shows a rapid change between the walking and jogging; therefore, three categories were defined for the regression analysis [32]. A one-sample t-test was then used to check whether the slope of the regression line was significantly greater than zero.

## 3. Results

**Figure 2:**
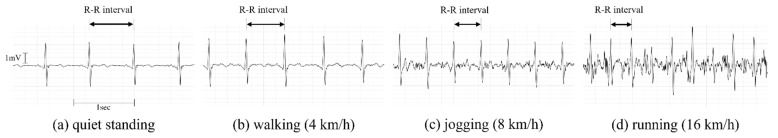
ECG waveforms recorded by the sportswear-type wearable: (**a**) at rest (quiet standing); (**b**) walking; (**c**) jogging; (**d**) running.

**Figure 3:**
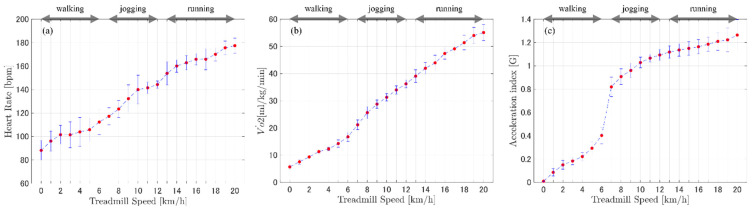
Mean and standard deviation of (**a**) heart rate (bpm), (**b**) oxygen uptake (${\dot{\mathrm{Vo}}}_{2}$), and (**c**) acceleration index at each treadmill speed across all participants.

**Figure 4:**
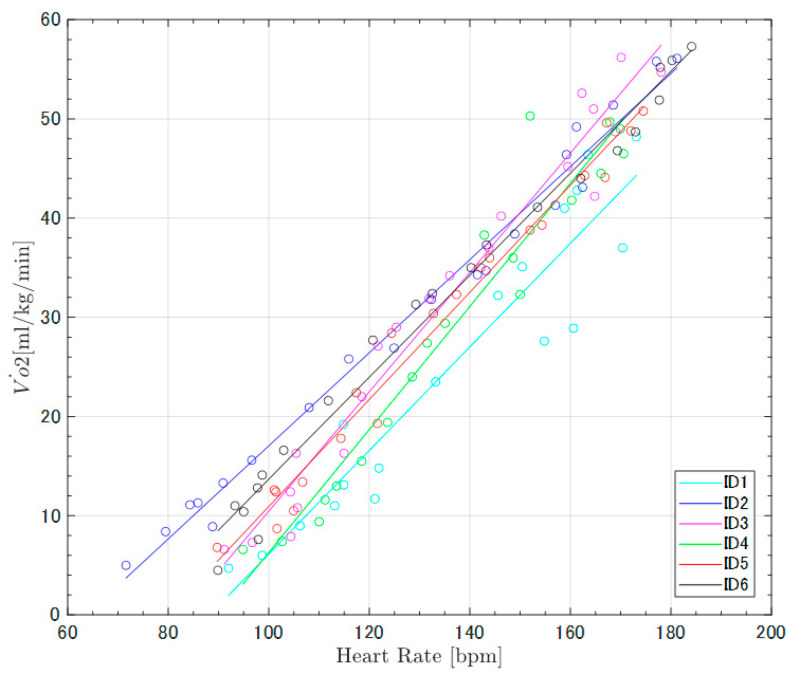
Regression lines of the heart rate and oxygen uptake comparison of all participants. All participants showed a linear relationship with small variations (r ≥ 0.96).

**Figure 5:**
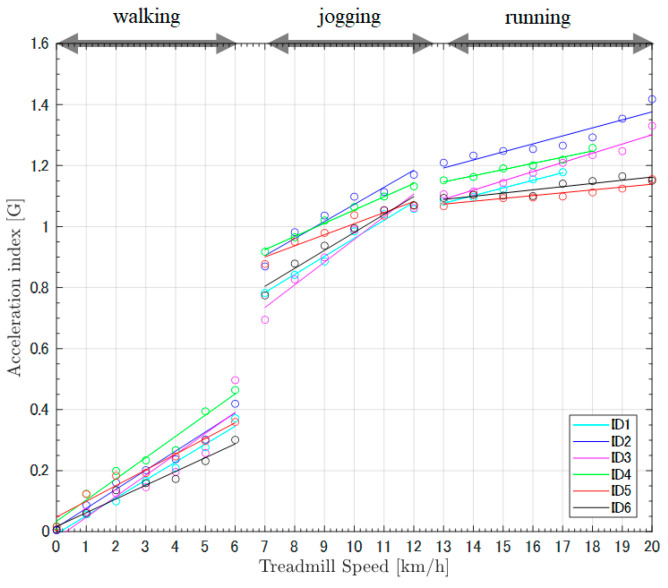
Regression lines of the acceleration index and treadmill speed comparison of all participants. All participants showed linear trends between acceleration index and treadmill speed within each condition: walking (0–6 km/h); jogging (7–12 km/h); and running (13–20 km/h).

The ECG signals obtained from the sportswear-type wearable were capable of detecting the R wave necessary for calculating the heart rate over a wide range of exercise intensities: low (walking), moderate (jogging), and high (running) (Figure 2). The physiological indices (heart rate and oxygen uptake) showed linear trends with increasing treadmill speed. Similarly, the acceleration index showed linear trends for all exercise intensities (Figure 3). However, a rapid change was observed at the switching point between low-intensity exercise (walking) and moderate-intensity exercise (jogging) (Figure 3). The single regression analysis between heart rate and oxygen uptake showed that the slope of the regression line was significantly greater than zero (*p* < 0.001), with a high coefficient of determination (R² = 0.96) (Table 1, Figure 4). Another single regression analysis between treadmill speed and the acceleration index showed that the slope of the regression line was significantly greater than zero at all intensities (*p* < 0.001) (Table 1, Figure 5), with a high coefficient of determination (walking, 0.95; jogging, 0.96; running, 0.90) (Table 1).

## 4. Discussion

We examined the feasibility of evaluating exercise intensity across a wide range of physical activity (quiet standing, walking, jogging, and running) using a sportswear-type wearable in a controlled laboratory setting. The results of this study demonstrated that (1) the heart rate recorded by the sportswear-type wearable highly correlated with the oxygen uptake, and (2) the acceleration index systematically increased alongside the rise in exercise intensity. We suggest that it is feasible to use the sportswear-type wearable to evaluate exercise intensity across different physical activities, and our results may be applicable to similarly designed wearables. In addition to the existing watch- and chest-belt-types, the sportswear-type wearable will be expected to contribute as a new device to increase the variety of wearables possible for monitoring exercise intensity during competitive matches and daily practice for athletes.

The sportswear-type wearable allows the detection of R-R intervals on the recorded ECG waveforms during quiet standing, walking, jogging, and running (Figure 2). This suggests that heart rate can be determined from the sportswear-type wearable, and the heart rate is an appropriate physiological indicator of exercise intensity across activities. The trend of increased artifacts during moderate-to-high-intensity movements such as jogging and running was also visually observed (Figure 2). The artifacts may be a result of the wearable’s motion artifact and contaminated muscle activity. Recently, wearable sensors have been used to predict and detect cardiac diseases [33]. With the current accuracy of ECG measurements by the sportswear-type wearable, it is not suitable to detect arrhythmia during high intensity exercise. However, it is assumed that it may be possible at rest and low-intensity exercise. In future studies, it is necessary to develop sportswear-type wearables that can detect arrhythmia during high-intensity exercise and sports activities.

The ECG-derived heart rate demonstrated a positive trend with increasing exercise intensity (Figure 3 and Figure 4). Although the validity of the ECG recording could not be evaluated by comparing its results with other ECG recording devices, the strong association between heart rate and oxygen uptake identified in this experiment indicates that the heart rate obtained from the sportswear-type wearable is adequate for evaluating physiological intensity. Indeed, prior studies have also reported a linear relationship between heart rate and oxygen uptake [34,35], reinforcing the validity of the heart rate recorded by our sportswear-type wearable. Currently, the sportswear-type wearables demonstrate an acceptable level of accuracy for detecting R-R intervals, indicating that the calculated heart rate has corresponding accuracy. A higher accuracy of ECG-derived heart rate recorded by the chest-belt-type wearable than photoplethysmography-derived heart rate recorded by the watch-type has been reported [23,24,25]. The ECG-derived heart rate recorded by the sportswear-type wearable has almost the same accuracy as the chest-belt-type wearable. However, the method of wearing is completely different, being strap-on and clothed. Therefore, the sportswear-type wearable can be a technology to relieve discomfort in chest-belt-type wearables due to the wrapping around the chest.

The linear association between the acceleration index and controlled treadmill speeds (walking, jogging, and running) (Figure 3 and Figure 5) demonstrates that the acceleration index recorded by the wearable’s inertial sensor is adequate for detecting changes in physical intensity. A previous study had reported a change in acceleration index when going up to a speed of 16.5 km/h; conversely, our experiment’s participants reached a greater speed (up to 20 km/h), which is common to many sports activities. In a previous study, the rapid change of acceleration index during the transition from waking to jogging was recorded by both the inertial sensor located on the upper back and the wearable’s sensor on the lower back [36,37]. The regression line’s slope was smaller during the running condition than during walking and jogging. This demonstrates that the sensitivity of the acceleration index relative to the change in activity intensity may slightly decrease when the athlete reaches running and sprinting intensity. However, the slope of the regression line was significantly greater than zero; therefore, the results suggest that the acceleration index recorded by the inertial sensor located on the upper back is useful for the evaluation of varied intensity in sports activities. GPS-recorded moving velocity has been widely used as an indicator of exercise intensity during competitive sports, especially team sports [38,39]. However, GPS validation studies have reported that the technology’s reliability and accuracy significantly reduce when individuals move fast over short distances, regardless of sampling frequency [40,41,42]. In addition, GPS devices are relatively large and heavy when compared with the accelerometers. Therefore, the small and light accelerometer on the upper back not only demonstrates feasibility for detecting fast-moving activity over a short distance but also causes less discomfort, making the wearable device adequate for competitive sports.

This study had two major limitations. First, we could not directly compare ECG results between those obtained from the sportswear-type wearable and the ground truth measure. Instead, we verified the feasibility of heart rate as an indicator of physiological intensity by external comparison with oxygen uptake. In future studies, direct comparisons of ECG records will provide results that better indicate the possibility of using sportswear-type wearables to detect abnormal ECGs derived from cardiovascular disease. Second, this study only tested a sportswear-type wearable designed for men. However, if a sportswear-type wearable designed for women adopts the same structural locations for the ECG electrodes and inertial sensor, we believe that a similar study to ours with female participants will also demonstrate feasibility for evaluating exercise intensity.

## 5. Conclusions

The present results showed that the heart rate recorded by the sportswear-type wearable highly correlated with the oxygen uptake in the exercise intensity from rest to high-intensity, and the acceleration index systematically increased alongside the rise of exercise intensity from rest to high-intensity. We suggest that the sportswear-type wearable with two integrated ECG electrodes and the inertial sensor is adequate for evaluating physical and physiological exercise intensity across the range from rest to high-intensity. In addition to existing wearables, the sportswear-type wearable can be used as one of the wearable options possible for monitoring exercise intensity during competitive matches and daily practice for athletes. The sportswear-type wearables will be used as one of the assistive devices to ensure sports-related safety and improve athletic performance.

## Figures and Tables

**Table 1:** Slope of regression lines and coefficient of determination.

	Acceleration Index and Treadmill Speed			Heart Rate and ${\dot{Vo}}_{2}$
	Walking	Jogging	Running	
Slope of regression line	0.06 (0.05–0.07)	0.06 (0.04–0.08)	0.02 (0.01–0.03)	0.54 (0.47–0.62)
Coefficient of determination of regression line (R^2^)	0.95 (0.86–0.99)	0.96 (0.93–0.99)	0.90 (0.77–0.99)	0.96 (0.90–0.99)

## Data Availability

The data analyzed in this manuscript will be made available from the corresponding author upon reasonable request.
